# Untargeted Metabolomics and Liquid Biopsy Investigation of Circulating Biomarkers in Soft Tissue Sarcoma

**DOI:** 10.3390/cancers17030553

**Published:** 2025-02-06

**Authors:** Daniela Grasso, Barbara Marzocchi, Guido Scoccianti, Ilaria Palchetti, Domenico Andrea Campanacci, Lorenzo Antonuzzo, Federico Scolari, Serena Pillozzi, Andrea Bernini

**Affiliations:** 1 Department of Biotechnology, Chemistry and Pharmacy, University of Siena, Via A, Moro 2, 53100 Siena, Italy; daniela.grasso@student.unisi.it (D.G.);; 2 Department of Orthopaedic Oncology and Reconstructive Surgery, Careggi University Hospital, Largo Giovanni Alessandro Brambilla 3, 50134 Florence, Italy; 3 Department of Chemistry “Ugo Schiff”, University of Florence, Via della Lastruccia 3, 50019 Florence, Italy; 4 Department of Health Science, University of Florence, Viale Pieraccini 6, 50139 Florence, Italy; 5 Department of Experimental and Clinical Medicine, University of Florence, Largo Brambilla 3, 50134 Florence, Italy; 6 Department of Experimental and Clinical Biomedical Sciences “Mario Serio”, University of Florence, Viale Morgagni 50, 50134 Florence, Italy

**Keywords:** soft tissue sarcoma, metabolomics, nuclear magnetic resonance, liquid biopsy, serum biomarkers, machine learning, purine salvage, ketone bodies, choline metabolism

## Abstract

Background: Soft tissue sarcomas (STSs) are rare, highly malignant mesenchymal tumours, comprising approximately 1% of all adult cancers and about 15% of paediatric solid tumours. STSs exhibit considerable genomic complexity with diverse subtypes, posing significant clinical challenges. Objectives: This study aims to characterise the molecular signature of primary STS through liquid biopsies and the untargeted metabolomic profiling of 75 patients, providing deep insights into cellular processes and potential therapeutic targets. Methods: This study analysed serum samples using nuclear magnetic resonance (NMR) spectroscopy for metabolomic profiling. Multivariate data analysis and machine learning classifiers were employed to identify biomarkers. Results: A panel of eleven significant deregulated metabolites were discovered in serum samples of patients with STS, with potential implications for cancer diagnosis and treatment. Conclusions: Choline decrease emerged as a marker for cancer progression, highlighting the potential of targeting its metabolism for therapeutic approaches in STS. The NMR analysis protocol proved effective for determining circulating biomarkers from liquid biopsies, making it suitable for rare disease research.

## Simple Summary

Soft tissue sarcomas are rare tumours with various subtypes, which makes it challenging to define their epidemiological characteristics. Treatment options are limited, with surgery as the primary approach, while the effectiveness of chemotherapy remains uncertain. This study aims to provide a stronger foundation for therapeutic research on this malignancy by characterising its molecular signature through liquid biopsies and untargeted serum metabolomics. A reduction in choline concentration was identified as a marker for cancer progression, indicating a potential therapeutic metabolic pathway target. The study also highlights the efficacy of nuclear magnetic resonance in profiling circulating biomarkers.

## 1. Introduction

Soft tissue sarcomas (STSs) are rare, highly malignant mesenchymal tumours, comprising approximately 1% of all adult cancers and about 15% of paediatric solid tumours [1,2,3]. STSs arise from mesenchymal tissues and exhibit diverse clinical presentations and molecular characteristics. In Europe, the incidence rate stands at 4.7 cases per 100,000, while in China, it is reported to be 2.9 cases per 100,000 [3,4]. STSs often manifest as painless enlarging masses arising from various anatomical sites throughout the body. The extremities are the most common location, accounting for approximately 60% of cases. Other frequently affected sites include the trunk (25%), retroperitoneum (15%), and head and neck region (5%).

Histologically, STSs encompass over 70 distinct subtypes, each with unique morphological features and biological behaviours. Moreover, molecular profiling studies have identified recurrent genetic alterations and signalling pathway dysregulations underlying various STS subtypes, further refining their classification and guiding targeted therapeutic approaches. The diverse pathological subtypes, combined with the low incidence rate, make it challenging to describe the epidemiological characteristics of STS, such as the incidence trend, age at diagnosis, prognosis, and metastasis risk.

At the molecular level, STSs exhibit considerable genomic complexity, characterised by chromosomal rearrangements, copy number alterations, and somatic mutations affecting oncogenes, tumour suppressor genes, and genes involved in DNA repair and cell cycle regulation. For instance, chromosomal translocations resulting in fusion oncogenes are hallmark events in several subtypes, such as EWSR1-FLI1 fusion in Ewing sarcoma and SS18-SSX fusion in synovial sarcoma.

Furthermore, the dysregulation of key signalling pathways, including the PI3K/AKT/mTOR pathway, MAPK pathway, Wnt/β-catenin pathway [5], and NER pathway [6], contributes to tumour growth, invasion, and metastasis in soft tissue sarcomas. The activation of receptor tyrosine kinases (RTKs), such as platelet-derived growth factor receptor (PDGFR), vascular endothelial growth factor receptor (VEGFR), and c-Met, is also commonly observed, driving angiogenesis and providing pro-survival signals to tumour cells [7].

Despite their relatively low incidence rate, STSs pose significant clinical challenges due to their heterogeneity, potential for aggressive behaviour, limited treatment options, and a 5-year survival rate of 50–70% [8]. In the past, STSs were almost always fatal diseases, and a significant portion of patients still die from distant metastases, occurring mainly in the lungs. Surgery continues to be the mainstay of treatment with the aim of wide or radical excision. Chemotherapy with different regimens has been adopted, but its efficacy is still to be proven in patients without evidence of metastatic disease. As with other rare tumours, soft tissue sarcomas can still be considered an ’orphan’ disease with preliminary studies, while extensive research is needed to develop new therapeutic options to improve patient survival further.

As for many cancer types, the selection of proper treatment in STS should rely on the presence of predictive tumour biomarkers. However, tissue biopsy, the most widely used method for classifying tumours and detecting biomarkers, provides information limited to a single point in space and time, making tumour heterogeneity one of the leading causes of therapeutic failure, particularly for histologically complex cases such as STS. An alternative to tissue biopsy is liquid biopsy, i.e., the sampling and analysis of non-solid biological tissue through rapid and non-invasive methods, with less discomfort for the patients and a faster recovery time, especially for those with tumours in difficult-to-reach areas. A blood-based liquid biopsy isolates and analyses tumour-derived or tumour-associated components circulating in the bloodstream: circulating tumour cells (CTCs), leukocytes, tumour-derived circulating nucleic acids, cancer-associated proteins, and dysregulated metabolites.

The latter are valuable biomarkers of rewired metabolic pathways playing a pivotal role in cancer progression, including STS. Indeed, tracking circulating metabolites over time allows for the frequent monitoring of tumour progression and the outcome of surgery, chemotherapy, relapse, and early detection of cancerous changes. It also provides a more comprehensive picture of cancer’s metabolic landscape, which can be missed with tissue biopsies that sample only specific parts of a tumour.

Cancer cells exhibit altered metabolism to sustain increased energy demands, support rapid proliferation, and adapt to the tumour microenvironment. Although metabolomics studies have already been widely used to identify diagnostic and prognostic biomarkers in other solid tumours, only a few targeted metabolic profiling of STS biofluids have been reported. Miolo et al. [9] considered the serum levels of 68 targeted metabolites, including 53 amino acids and their derivatives, as well as 15 bile acids, from 24 metastatic patients; Jia et al. [10] reported an LC/MS/MS investigation targeting plasma-free amino acid profiles (PFAAs) of 23 patients; Toulmonde et al. [11] observed a statistically significant increase in the kynurenine to tryptophan ratio between plasma samples upon the infusion of pembrolizumab in a cohort of 50 patients. The heterogeneous nature of STS further hampers these studies, which have a small number of samples.

Therefore, the present work aims to characterise the metabolomic signature of primary STS based on liquid biopsies and the untargeted metabolomic profiling of 75 patients, covering all the major histotypes. Metabolic profiling provides valuable information on biofluids [12,13,14,15], with serum often chosen for representing phenotypes in diagnosis, therapeutic follow-up, and systems biology studies. Unlike targeted approaches, untargeted profiling does not require predefined hypotheses about which metabolites are important. This makes it ideal for exploratory studies in which the goal is to identify unexpected or novel changes in metabolism. Moreover, untargeted metabolomics can provide deep insights into cellular processes and biological functions by capturing a broad spectrum of metabolites. It may reveal how various metabolic pathways interact and respond to different biological conditions. Profiling can be accomplished using nuclear magnetic resonance (NMR) spectroscopy, a highly reproducible and inherently quantitative experiment. NMR is non-destructive and requires minimal sample preparation. Thus, the sample is not altered or consumed during analysis and can be preserved for further testing. Moreover, NMR data are relatively straightforward to interpret, making NMR more suitable for an untargeted approach compared to other techniques, which often require prior knowledge or extensive database searching for metabolite identification. Also, NMR is very effective at detecting very-low-molecular-weight compounds common in metabolomics studies because of their sharp spectral peak, e.g., acetate and acetone. The use of intact serum is also possible following a protocol recently developed at our lab with minimal modifications [16]. While the method’s advantages and the large cohort studied have led to new findings, STS metabolomics remains challenging. Its high degree of heterogeneity prevents histotype stratification based on metabolic profiling, and the data from the overall analysis are reported. Therefore, further subtype-specific studies are necessary to develop a precision medicine therapeutic approach for STS [17].

## 2. Materials and Methods

### 2.1. Patients with STS and Sample Preparation

Patients with STS who were diagnosed with STS after an accurate revision by an expert pathologist were eligible for inclusion in the study. Both adult and paediatric patients were included. Exclusion criteria were a diagnosis of different tumours and a lack of consent to participate in the study.

Serum was obtained from peripheral blood collection followed by separation and centrifugation, then stored at −80 °C until NMR sample preparation. Samples for NMR measurement were prepared with 300 µL of serum, 260 µL TRIS-d11 buffer 150 mM (final concentration of 65 mM), and 40.0 µL formate 45.0 mM (final concentration of 3.0 mM). The total deuterium concentration in the samples was maintained at 10% for NMR purposes. Tris-d11 (Tris (hydroxymethyl-d3)amino-d2-methane), calcium formate, and D_2_O were purchased from Sigma-Aldrich, St. Louis, MI, USA.

### 2.2. ^1^H NMR Spectroscopy

All experiments were performed on a Bruker Avance III™ 600 spectrometer operating at 14.1 T. The PROJECT pulse sequence [18,19,20] was used with an echo time (τ) of 0.3 ms and 128 loops to obtain the optimised T2 filter delay [16]. All spectra were obtained with 32 scans, each with a spectral width of 6 kHz, digitalised over 32k points and zero-filled to 256k points. Solvent signal removal was achieved with a presaturation power of 43 dB during the repetition delay (4 s).

### 2.3. ^1^H NMR Data Processing

The NMR data were processed and analysed with Chenomx 10 (Chenomx. Edmonton, AB, Canada). Formate was used as an internal standard to calibrate the other metabolites’ concentrations. We used the Chenomx library to identify and quantify a total of 63 metabolites (see Table S1 in Supplementary Materials).

### 2.4. Data Analysis

The principal component analysis was carried out with Metaboanalyst 6.0 [21]. The random forest (RF) classifier followed the workflow from Huang et al. [22]. First, we drew bootstrap samples from the original data to create a random forest (RF) model with 2000 trees. Then, for each of these samples, we grew an unpruned classification or regression tree, but instead of considering all variables for the best split at each node, we randomly selected a subset of the variables and chose the best split from those variables. Finally, to make predictions for new data, we aggregated the predictions of all 2000 trees, which essentially involved taking a majority vote for classification tasks.

## 3. Results

### 3.1. Demographic and Clinical Characteristics of the Patients and Healthy Controls

A total of 75 serum samples were obtained from patients with STS enrolled at the Oncology and Reconstructive Orthopedics SOD, AOU Careggi (RESEARCH study), after they provided their written consent. The main clinical and pathological characteristics of the patients are summarised in Table 1. The STS serum samples were used for NMR sample preparation as explained in the Methods section. Serum samples of age and sex-matched controls in healthy conditions and with no metabolic disorders were also analysed similarly (*n* = 85; 56% male and 44% female; mean age = 62; age max = 95; age min = 32). All STS samples and control samples were used for the analysis.

### 3.2. Nuclear Magnetic Resonance Metabolomic Profiling

**Figure 1:**
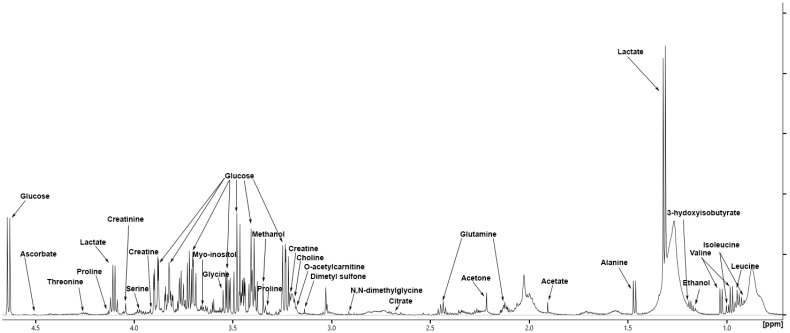
Hydrogen 1D spectrum (aliphatic region) of serum sample from an STS patient. Signals of some relevant metabolites among the sixty-two identified are labelled. The lower limit of detection (LoD) was estimated to be 1 µM.

Metabolic profiling offers valuable insights into biofluids. Among them, serum is the most commonly selected for diagnosing phenotypes, monitoring treatment progress, and conducting systems biology studies. This profiling can be carried out using NMR spectroscopy in a non-destructive and highly reproducible manner, whether using a targeted or untargeted approach. However, it is essential to optimise sample preparation and experimental conditions to ensure the reliability of subsequent statistical analyses. We applied NMR metabolomic profiling in an untargeted fashion using the PROJECT pulse sequence [18,19,20] to intact serum samples according to a previously reported protocol [16]. Software-assisted deconvolution using Chenomx 10 (Chenomx. Edmonton, AB, Canada) allowed for 63 metabolites to be identified and quantified as follows (Figure 1): glucose, lactate, urea, glycine, alanine, glutamate, glutamine, proline, acetate, valine, ethanol, threonine, serine, glycerol, 3-hydroxybutyrate, taurine, leucine, acetone, histidine, lysine, citrate, mannose, isoleucine, betaine, aspartate, carnitine, tyrosine, creatine, 2-hydroxybutyrate, creatinine, ornithine, mannitol, myo-inositol, methanol, choline, phenylalanine, 2-aminobutyrate, asparagine, arabinose, dimethyl sulfone, pyruvate, o-acetylcarnitine, isopropanol, methionine, propylene glycol, n,n-dimethylglycine, isobutyrate, dimethylamine, trimethylamine n-oxide, 2-hydroxyisovalerate, 2-oxoisocaproate, 3-hydroxyisovalerate, 3-phenylpropionate, acetoacetate, acetoin, ascorbate, hypoxanthine, inosine, sucrose, xanthine, succinate, π-methylhistidine, and τ-methylhistidine. The observed metabolites span several molecular classes: amino acids, amines, carboxylic acid, ketones, thiones, alcohols, nucleobases, nucleosides, and carbohydrates. However, four metabolites were excluded from the analysis because they are drug constituents (mannitol), lab equipment pollutants (ethanol and isopropanol), or were unreliably quantified by NMR, such as urea [23] (see Table S1 in Supplementary Materials).

### 3.3. Multivariate Data Analysis and Machine Learning Classifiers

**Figure 2:**
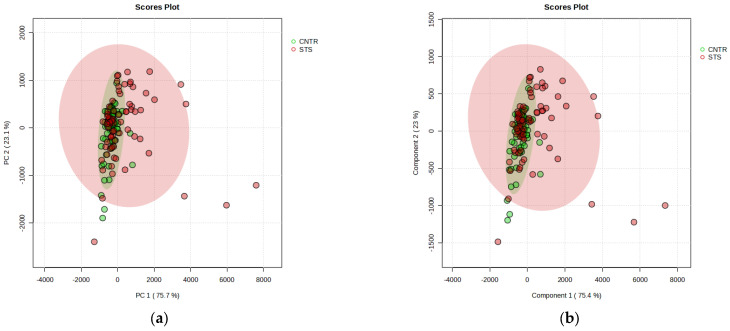
PCA (**a**) and PLS-DA (**b**) scores plot of patients with STS (red) vs. healthy controls (green). Intra-group variation in patients is broad compared to the minimal variation in controls.

Serum metabolite levels were subjected to a principal component analysis to categorise the patients and controls. The distribution of the two groups was presented by plotting the first two principal components (Figure 2a).

The PCA score plot overlaps the two groups, indicating that the unsupervised PCA method failed to extract meaningful insights from the NMR data. We also applied a partial least squares discriminant analysis (PLS-DA) to classify the two groups, as illustrated in Figure 2b. PLS-DA, a supervised technique, reduces dimensionality like a PCA but aims to maximise class discrimination within the latent space. Despite using this approach, a significant overlap remains between the sets. The challenges faced by both PCA and PLS-DA in analysing data from such a heterogeneous STS group may stem from their reliance on linear dimensionality reduction methods. When the relationships within the data are nonlinear, these methods may struggle to differentiate the groups. To overcome these challenges, alternative nonlinear techniques, such as machine learning models, can be utilised to identify complex, nonlinear relationships between features and target variables. Then, the random forest (RF) method was used to explore the underlying characteristics of these NMR data.

The RF method leverages two powerful machine learning techniques: bagging and random feature selection. Bagging involves training each tree on a bootstrap sample of the training data and making predictions using a majority vote of trees. Additionally, the RF model randomly selects a subset of features to split at each node when growing a tree rather than using all features. To evaluate prediction performance, the RF algorithm uses out-of-bag (OOB) samples in a type of cross-validation that runs parallel to the training step. Each tree is grown on average using about two-thirds of the training data, leaving one-third as OOB samples. Because the OOB error is calculated using out-of-bag samples, which are not used in the model’s training, it provides an unbiased estimate of its performance. In our biomarker discovery study, the RF model can also estimate the importance of features during training. This is achieved by measuring how each feature contributes to the prediction performance of the RF algorithm. The calculation involves determining the mean decrease in classification based on permutation. In other words, it subtracts the prediction accuracy after permutation from the prediction accuracy before permutation. Then, it averages the result for all trees in the forest to obtain the value of the permutation importance. This value is then used to identify biomarkers.

**Figure 3:**
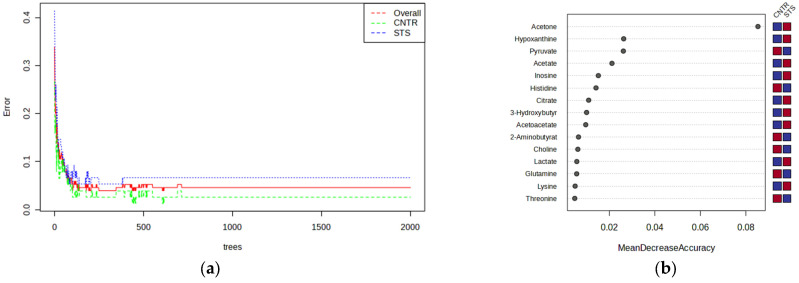
(**a**) Plot of OOB error for RF classification of the two groups; overall OOB estimate is 0.04. (**b**) Mean decrease in accuracy indicates the features most relevant to the model; red and blue squares indicate higher and lower concentrations, respectively.

We then employed the RF method to explore the important features of the NMR analysis. Two thousand trees were grown, and the OOB estimate of the error rate and a five-fold cross-validation (CV) were used to evaluate the stability of the forest tree model. Figure 3 shows that the OOB error rate stabilised with the number of trees constructed to a value of 4%, indicative of good performance; a similar value from CV indicates the model is not overfitting the data.

### 3.4. Biomarker Discovery

Metabolic biomarker discovery is a key goal of metabolomics studies. During model construction, variable selection aims to identify the optimal combination of variables for the best classification results [22]. The mean decrease in classification based on permutation was used as a variable importance measure (VIM). Then, a *t*-test was implemented to test the significance of these metabolites, and the results for *p* < 0.01 are summarised in Table 2. Eventually, we obtained 11 significantly deregulated metabolites related to the STS, including acetone, hypoxanthine, inosine, acetate, histidine, choline, 3-hydroxybutyrate, citrate, acetoacetate, lactate, and pyruvate.

As mentioned in the introduction, limited studies have conducted metabolic profiling of STS biofluids, with observations mostly limited to amino acids. In this regard, our research identified a significant downregulation of histidine in the serum of patients with STS, echoing findings suggested by a PCA analysis for a small subset of patients in Miolo et al.’s study [9]. Our machine learning method validated that this metabolite is among the key contributors to the alteration of the metabolomic profile in STS compared to the control at baseline.

Our NMR untargeted approach enabled the exploration of various other molecular classes, broadening the metabolic profiling to include amines, short-chain fatty acids, ketones, nucleobases, carbohydrates, and sulphur compounds. This was conducive to novel findings, such as the downregulation of choline (Table 2), which we also suggest is a circulating biomarker of tumour progression following the overall survival analysis (see Section 4.2 for details) [24,25].

The other contributing metabolites are first reported as circulating biomarkers in STS, although they were expected because of their long record in cancer research, as described below.

Ketone bodies have been reported as not efficiently utilised by tumour cells for mitochondrial dysfunction and/or deficient ketolytic enzymes [26,27,28,29], a condition known as acquired metabolic inflexibility [30]. Our results support this condition, indicating an increase in ketogenesis.

Increased levels of the circulating hypoxanthine and inosine are reported. In healthy controls, purines are below the detection limit, while in STS, inosine and hypoxanthine, but not the catabolic product xanthine, can be found up to the tens of micromolar range. Such increased levels can be ascribed to cell stress, such as hypoxia or apoptosis, and fuel the purine salvage pathway.

Like purines and ketone bodies, serum levels of acetate and citrate are elevated in the patients with STS analysed in our study, indicating a favourable environment for uptake by cancer cells to support biomass production.

Lactate and pyruvate form an axis that is well known in cancer and are described in detail in Section 4.1 under the Warburg effect; their counter-regulation was exploited by introducing the new feature, the lactate to pyruvate ratio, showing a much-improved significance.

The correlation between dysregulated metabolites and clinical and demographic features was pursued without significant results, potentially hindered by intrinsic tumour heterogeneity.

The Discussion section explores pathways related to these metabolites and their possible function as biomarkers.

## 4. Discussion

### 4.1. Biomarkers and Pathways

#### 4.1.1. Ketone Bodies and Metabolic Inflexibility

**Figure 4:**
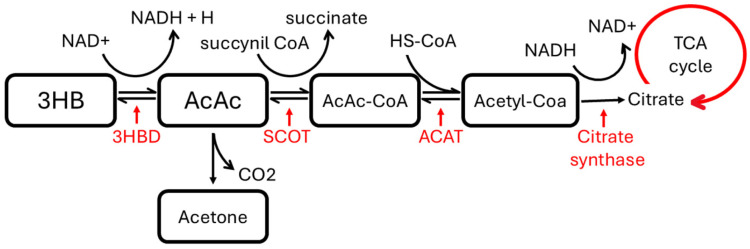
Acetoacetate (AcAc), provided by 3-hydroxybutyrate (3HB) dehydrogenation by 3-hydroxybutyrate dehydrogenase (3HBD), can be either converted to acetone through non-enzymatic decarboxylation or to acetyl-CoA. Indeed, AcAc is first converted by succinyl-CoA:3-ketoacid CoA transferase (SCOT) to acetoacetyl-CoA (AcAc-CoA), the substrate of acetoacetyl-CoA thiolase (ACAT). Acetyl-CoA goes through the citric acid cycle and, after oxidative phosphorylation, produces 12 ATP per molecule. Acetone is not re-converted to acetyl-CoA, which is either excreted through urine or exhaled.

Ketone bodies (KBs) are water-soluble molecules produced in the liver as byproducts of fat metabolism; the three main types of ketone bodies are acetoacetate (AcAc), 3-hydroxybutyrate (3HB), and acetone (Ace). Cells efficiently convert ketone bodies into acetyl-CoA, which is then released into the TCA cycle to generate energy and enhance cell viability under low-carbohydrate conditions (Figure 4) [31]. The most degraded molecule, acetone, is the most upregulated within the panel.

#### 4.1.2. Acetate, Citrate, and Fatty Acid Synthesis

In adult humans, new fatty acid (FA) production primarily occurs in the liver, adipose tissue, and lactating breast [32]. Alternatively, increased FA production may result from cancer cells’ high metabolic needs or an adjustment to the limited availability of lipid molecules derived from the bloodstream in the tumour microenvironment. Two major players in FA synthesis are acetyl-CoA (AcCoA) and citrate. Due to their role in biomass production, cancer cells are in high demand for AcCoA [33]. In conditions with plenty of nutrients and oxygen, this demand is met chiefly by producing AcCoA from glucose carbon in the mitochondria. The AcCoA is then used to generate citrate, which is transported to the cytosol to produce cytosolic AcCoA. However, in hypoxic conditions, most glucose is directed towards lactate production, leading to decreased AcCoA production from glucose [34]. Recently, a third substrate, acetate, has been found to support AcCoA synthesis [35], particularly in hypoxic and highly glycolytic tumour cells [33]. Studies have also revealed that cancer cells show increased acetate uptake under hypoxia, even in low acetate concentrations, to support tumour growth [36,37,38]. Moreover, tumour cells may increase extracellular citrate uptake to sustain fatty acid synthesis rather than relying on the Krebs cycle [39].

#### 4.1.3. Choline

Choline metabolism is crucial for various cell functions, including cell membrane formation, single-carbon metabolism, and cholinergic neurotransmission [40]. It is a key component of phosphatidylcholine (PtdCho), a primary element of cell membranes [41]. It can be derived from food or synthesised within our bodies [42]. Recent research indicates that cancer increases the demand for choline and its metabolites [43]. Indeed, the rapid growth of cancer cells requires the increased production of PtdCho to form new cell membranes [44,45]. Furthermore, altered signalling pathways in cancer cells enhance the uptake and utilisation of choline. In our cohort of patients with STS, we observed a significant decrease in the median serum choline levels compared to the serum levels of healthy donors. This suggests an increased uptake necessary to support membrane production in developing tumour tissue.

#### 4.1.4. Inosine, Hypoxanthine, and Purine Salvage Pathway

Inosine and hypoxanthine are essential intermediates in the purine salvage pathway, a metabolic route that recycles purines to synthesise nucleotides. The purine salvage pathway is essential for recycling purine bases to synthesise nucleotides without de novo synthesis, which is energetically expensive. Tumours use such circulating precursors to supply the purine salvage pathway and gain purine nucleotides [46].

**Figure 5:**
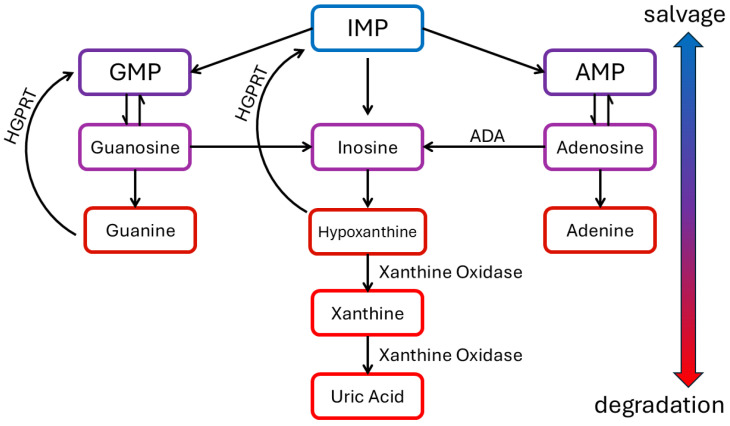
Purine salvage pathway. In healthy people, inosine is usually degraded into uric acid and excreted in urine. However, in STS, there are high concentrations of inosine and hypoxanthine, suggesting tumour cells are not capable of de novo synthesis due to their hypoxic state and salvage can be exploited. Inosine and hypoxanthine are then re-converted into purine nucleotides.

**Figure 6:**
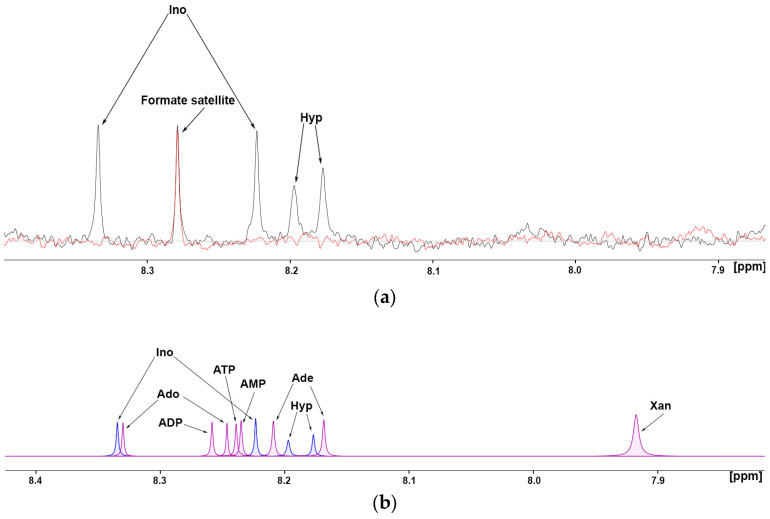
(**a**) Purines’ aromatic ring region of the NMR spectrum from an STS serum (black) shows the typical signals of inosine (Ino) (29 µM) and hypoxanthine (Hyp) (25 µM); such signals are absent in all control spectra (red) down to the lower limit of detection (1 µM). Satellite peaks from formate arise in this area due to residual 13C coupling and are to be ignored. (**b**) Simulated peaks from purine rings: adenosine (Ado), adenine (Ade), xanthine (Xan), inosine (Ino), hypoxanthine (Hyp), and adenosine nucleotides (AMP, ADP, ATP). Large peak dispersion in such spectral region (7.8–8.4 ppm) is apparent, making purines effectively identifiable and quantifiable by NMR. Patients show remarkable Ino and Hyp levels (blue) but not the other purines (pink).

Hypoxanthine is a key intermediate in the pathway. It can be salvaged to form inosine monophosphate (IMP) by the enzyme hypoxanthine-guanine phosphoribosyltransferase (HGPRT). IMP can then be converted into other nucleotides, such as adenosine monophosphate (AMP) and guanosine monophosphate (GMP), that are essential for DNA and RNA synthesis. Inosine is formed from the deamination of adenosine by adenosine deaminase (ADA, epithelial, and peripheral membrane protein), transferred into the cell by equilibrative nucleoside transporters (ENTs) [47,48,49,50,51] and converted back to hypoxanthine by the enzyme purine nucleoside phosphorylase (PNP and cytosolic), closing the circle to purine salvage. Circulating hypoxanthine can be transported into the cytosol by ENT2 as well [52]. Hypoxanthine (Hyp) can be oxidised to xanthine by xanthine oxidase. Xanthine (Xan) is not directly reused in nucleotide synthesis but is further oxidised to uric acid, which is excreted from the body. The process is fuelled by extracellular adenosine nucleotides (ATP, ADP, and AMP) produced/released during stress states such as hypoxia or cell damage and dephosphorylated by a step-wise process involving a series of ectonucleotidases [53,54,55]. This process leads to increased concentrations of circulating adenosine and its degraded purines (Figure 5). This scenario may happen at sites where oxygen demand exceeds oxygen supply, such as the tumour microenvironment affected by inflammatory cytokines [56]. Abnormalities in the circulating purine levels (e.g., adenosine nucleotides, adenosine, inosine, hypoxanthine, and xanthine) can be easily monitored by NMR as their aromatic rings give sharp resonances in a non-overlapped spectral region (Figure 6a).

Such behaviour also lowers their limit of detection to <1 μM. In healthy controls, purines are below the detection limit, while in STS, inosine and hypoxanthine, but not the catabolic product xanthine, can be found up to the tens of micromolar range. The results can be ascribed to cell stress, such as hypoxia or apoptosis.

#### 4.1.5. Histidine

A growing body of research suggests that the levels of histidine (His) found in the bloodstream of cancer patients are altered compared to those in healthy individuals. As the source of one carbon unit, histidine supplies the formimino group to tetrahydrofolate, which participates in the synthesis of purines and pyrimidines [57]. It is suggested that the overactive nucleic acid metabolism in tumour cells leads to the excessive absorption of histidine into tumour tissue, resulting in increased consumption and lower levels in the body [58].

Interestingly, in our samples, the histidine concentrations decrease to an average of 35 µM, which aligns with the 30 μM value already associated with lung cancer progression [59,60,61].

#### 4.1.6. Lactate, Pyruvate, and the Warburg Effect

**Figure 7:**
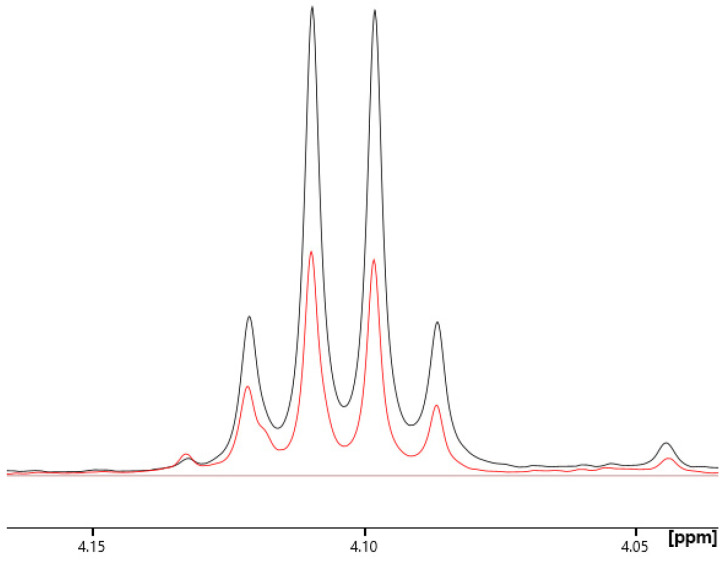
Comparison of peak area, proportional to concentration, of lactate alpha-hydrogen signal between representative patient (black, C = 2.3 mM) and control (red, C = 1.1 mM). The large excess of lactate in the patient is apparent.

Cancer cells avidly consume glucose and convert it to lactate, resulting in a low pyruvate level. This phenomenon is known as the Warburg effect or aerobic glycolysis and is important for cell proliferation. The Warburg effect is a phenomenon in which cancer cells predominantly produce energy through a high rate of glycolysis followed by lactic acid fermentation in the cytosol rather than through the more energy-efficient oxidative phosphorylation in the mitochondria, even when oxygen is abundant. This shift allows cancer cells to proliferate rapidly by generating energy and the metabolic intermediates required for synthesising cellular components [62]. Our observations in the bloodstreams of patients with STS show Warburg activation, in which lactate is upregulated and pyruvate is downregulated, conducive to a lactate-to-pyruvate ratio exceeding that of the healthy controls by three-fold on average, an extent so large it can be visually estimated from NMR signals (Figure 7).

### 4.2. Association of Overall Survival with Clinical Features and Metabolic Profiles

At the checkpoint analysis, overall survival data were available for 60 patients, 18 of whom had died. The overall survival median was 29 months (95% CI 24–33) within a minimum of 6 months. Low-graded cases scored better, with a median of 35 months (95% CI 24–39) with a minimum of 21 months, while gender and age at diagnosis do not contribute to significant differences.

The quantitative metabolomics data concerning overall survival (OS) were analysed using a univariate Cox proportional hazards regression to link metabolite dysregulation with tumour progression. Choline demonstrates a *p*-value corrected for the false discovery rate significantly associated with OS (*q*-value < 0.05), as shown in Table 3. No clinical features were found to be significantly associated with choline levels.

## 5. Conclusions

Our untargeted metabolomics investigation of STS liquid biopsy allowed for quantifying metabolites representative of several molecular classes from 75 patients. This differs from previous studies on circulating biomarkers, which mainly target free serum amino acids.

The metabolic profiling revealed eleven dysregulated circulating metabolites. The Warburg effect significantly influenced the NMR spectra of the serum STS samples, increasing the lactate-to-pyruvate ratio. Some low-level metabolites, such as ketones and purines, were found to be upregulated; these metabolites serve as circulating fuels for the biosynthesis of lipids and nucleotides, addressing the heightened demand from tumour cells.

Choline decrease has emerged as a marker for cancer progression. Its significant downregulation in STS has important implications for understanding the metabolic preferences and adaptive strategies of cancer cells, highlighting the potential of targeting choline metabolism as a therapeutic approach in sarcoma types.

On the methodological side, the protocol for the NMR analysis of intact serum samples with minimal manipulation, time, and costs proved effective in determining circulating biomarkers from liquid biopsies, making it suitable for rare disease research.

Despite the metabolomic analysis, the significant heterogeneity of STS hindered patient stratification, and the findings pertain to the entire histotype dataset. Future studies concentrating on subtype-specific analyses are essential to address the lack of stratification and enhance the clinical relevance of circulating biomarkers.

## Figures and Tables

**Table 1:** Clinicopathological features of the patients enrolled with STS.

Clinicopathological Features	Cohort (*n* = 75)
Gender	Male = 56%
	Female = 44%
Age at surgery	Mean = 64
	Max = 92
	Min = 22
Primary/relapse	Primary = 85%
	Relapse = 15%
Grade	High = 72%
	Intermediate = 8%
	Low = 20%
Histotype	Pleomorphic sarcoma = 29%
	Liposarcoma = 25%
	Mixofibrosarcoma = 16%
	Leiomyosarcoma = 9%
	Sinovial sarcoma = 7%
	Rhabdomyosarcoma = 6%
	Dermatofibrosarcoma protuberans = 4%
	Fibrosarcoma = 4%
Anatomical site	Thigh = 65%
	Upper-limb = 11%
	Gluteal-sacral region = 9%
	Pectoral region = 5%
	Deltoid region = 3%
	Scapular region = 3%
	Abdominal region = 2%
	Foot = 2%

**Table 2:** A panel of metabolites from serum samples of patients with STS resulting in up- or downregulation (see arrows) compared to serum samples of healthy donors with a significance lower than 0.01 are reported alongside the relevant metabolic process.

Feature	*p*-Value	Up/Downregulation
Acetone	<0.00001	↑
Acetoacetate	<0.001	↑
3-Hydroxybutyrate	<0.00001	↑
Acetate	<0.00001	↑
Citrate	<0.0001	↑
Choline	<0.00001	↓
Hypoxanthine	<0.00001	↑
Inosine	<0.00001	↑
Histidine	<0.00001	↓
Lactate	<0.001	↑
Pyruvate	<0.01	↓
Lac/Pyr	<0.00001	↑

**Table 3:** Most significative metabolites from univariate Cox regression analysis. Choline shows a significantly low false discovery rate (*q*-value).

Feature	*p*-Value	*q*-Value ^a^
Choline	0.0008	0.0092
Acetate	0.0214	0.1236
Inosine	0.0697	0.2683
Hypoxanthine	0.1195	0.2770

^a^ False discovery rate calculated as q-value following Benjamini, Krieger, and Yekutieli’s procedure.

## Data Availability

Data is contained within the article or Supplementary Materials.

## Supplementary Materials

The following supporting information can be downloaded at: https://www.mdpi.com/article/10.3390/cancers17030553/s1, Table S1: Concentrations of serum metabolites from STS patients quantification in NMR, such as Urea. Metabolites with more than 50% of their values not available were also removed for clarity.
