# Supplementary material for: Untargeted Metabolomics and Liquid Biopsy Investigation of Circulating Biomarkers in Soft Tissue Sarcoma
Source: Cancers (Basel). 2025 Feb 6;17(3):553. doi: 10.3390/cancers17030553 (PMC11817886; doi:10.3390/cancers17030553)
Supplement: Supplementary file 1 [file cancers-17-00553-s001.zip › cancers-3407824-supplementary.pdf]

Table S1. Concentrations (μM) of serum metabolites from STS patients. Four metabolites were excluded from the analysis because they are drug constituents (Mannitol), lab equipment pollutants (Ethanol, Isopropanol) or had unreliable quantification in NMR, such as Urea. Metabolites with more than 50% of their values not available were also removed for clarity.

Pt.	2-Aminobutyrate	Acetate	3-Hydroxybutyrate	Acetoacetate	Acetone	Alanine	Asparagine	Aspartate	Betaine	Carnitine	Choline	Citrate	Creatine	Creatinine	Dimethyl sulfone	Dimethylamine	Glucose	Glutamate	Glutamine	Glycerol	Glycine	Histidine	Hypoxanthine	Inosine	Isobutyrate	Isoleucine	Lactate	Leucine	Lysine	Mannose	Methanol	Methionine	N,N-Dimethylglycine	O-Acetylcarnitine	Ornithine	Phenylalanine	Proline	Pyruvate	Serine	Succinate	Taurine	Threonine	Trimethylamine N-oxide	Tyrosine	Valine	myo-Inositol
1	17.2	35.0	38.8	6.2	27.0	286.4	45.0	86.0	33.8	33.2	23.8	43.4	13.8	22.0	24.0	3.6	273.4	135.2	266.8	99.4	278.0	62.2	32.4	0.0	3.4	55.0	4424.8	90.8	78.2	39.0	309.4	15.6	3.4	6.0	61.8	49.4	123.2	60.0	138.0	6.6	49.8	150.0	4.2	32.6	126.8	31.8
2	10.4	52.4	66.0	23.0	46.4	147.0	36.4	39.2	28.6	25.4	13.0	55.8	14.6	40.4	22.6	6.4	2134.8	25.2	360.2	78.6	125.2	46.6	0.0	0.0	3.0	47.8	718.4	67.6	75.2	59.8	76.6	13.4	4.0	2.8	33.0	32.4	124.6	10.0	67.6	3.4	49.4	121.8	1.8	30.6	115.0	28.4
3	15.6	22.6	40.6	15.2	35.0	262.4	30.8	45.6	17.4	31.2	17.8	49.6	23.4	40.0	21.0	4.0	2789.0	66.0	302.0	92.8	143.0	67.4	24.6	23.4	3.8	50.2	1819.8	91.6	76.0	52.0	128.8	17.6	2.4	4.0	53.6	36.6	130.4	10.6	89.6	1.8	51.0	107.2	3.4	47.6	171.0	29.4
4	7.8	25.6	49.2	34.2	18.8	237.8	35.6	47.0	16.4	37.2	6.4	41.2	32.6	38.2	16.6	3.6	2609.0	54.6	309.6	69.6	201.4	63.6	12.4	0.0	3.8	42.2	988.4	77.6	84.6	60.8	202.4	24.8	4.0	2.4	55.6	40.6	172.6	14.6	89.2	1.4	64.8	142.0	1.8	35.0	137.8	42.8
5	14.2	35.4	67.8	33.8	150.6	113.0	38.4	44.0	16.0	32.6	14.2	67.8	5.0	43.0	14.0	5.0	2171.8	30.0	310.0	86.6	145.6	29.8	11.6	0.0	2.6	37.4	579.4	59.8	74.2	86.4	72.6	15.2	4.6	6.4	36.4	29.2	84.2	6.0	78.4	2.7	42.8	85.6	5.4	28.6	92.8	29.6
6	30.6	53.4	90.8	37.0	48.0	204.8	35.8	31.0	18.4	37.8	15.2	40.6	13.8	39.0	13.2	3.6	2852.8	47.8	285.0	95.4	156.8	43.6	0.0	0.0	2.8	54.8	781.2	125.0	89.0	112.2	102.6	22.0	4.6	8.0	41.0	38.0	148.6	24.2	242.6	4.6	42.8	135.0	3.1	42.2	156.2	29.0
7	23.2	28.8	19.0	14.6	18.2	184.4	29.0	34.2	27.2	33.0	20.2	46.8	21.6	55.6	11.8	8.6	3073.0	38.6	288.0	55.2	192.6	77.8	0.0	0.0	3.0	31.2	552.4	71.6	69.4	60.8	148.4	14.8	4.0	6.2	38.0	22.2	148.8	4.0	84.0	3.0	41.0	114.4	3.4	31.6	122.6	32.4
8	21.2	196.4	218.2	62.0	127.8	213.4	37.8	44.0	18.6	26.6	11.8	35.6	12.8	34.4	11.4	6.0	2568.4	56.0	287.8	88.4	161.2	43.6	0.0	0.0	4.2	53.4	1060.4	89.8	62.2	80.4	71.4	15.8	5.2	5.0	22.2	23.4	130.2	9.0	63.0	1.6	42.8	115.0	3.8	26.0	156.6	29.4
9	4.8	63.3	72.9	17.1	88.8	106.5	24.9	38.8	21.3	30.6	11.7	18.0	11.7	52.5	11.4	3.6	1640.4	61.5	273.0	68.1	105.0	32.4	22.2	25.8	3.0	34.8	1428.0	57.9	61.2	38.1	88.5	16.5	4.5	6.6	30.0	45.3	114.0	8.1	56.1	4.8	58.8	64.5	5.1	31.2	100.2	51.0
10	9.6	58.8	22.4	14.6	8.0	201.8	43.4	38.8	30.6	60.0	11.7	30.8	11.6	24.6	11.0	5.0	2169.6	48.6	306.6	50.8	148.2	53.2	0.0	0.0	1.6	36.6	674.8	66.2	61.8	36.4	164.6	15.6	2.4	5.1	21.2	22.4	154.0	6.4	63.2	3.0	30.8	104.8	2.4	27.6	127.8	19.0
11	22.2	77.0	186.0	136.4	35.2	107.4	18.6	46.4	6.6	15.8	4.4	25.6	24.8	28.6	9.8	9.2	3125.6	61.6	198.6	78.8	111.6	37.2	16.8	9.8	5.4	88.6	1201.8	149.4	62.4	111.4	233.2	15.4	3.0	3.8	40.8	40.6	101.0	6.4	68.4	3.4	24.2	102.4	1.2	31.6	200.2	30.0
12	16.2	26.2	48.8	17.2	19.0	196.4	29.0	61.6	26.8	41.2	21.4	37.4	46.6	20.4	9.4	5.8	3243.6	58.0	309.0	94.8	235.6	56.0	0.0	0.0	3.8	26.8	687.0	42.8	52.6	49.8	147.2	14.4	4.4	9.6	16.0	24.2	162.2	22.6	104.2	3.2	42.8	140.4	10.6	21.4	95.0	25.8

myo-Inositol	21.2	32.0	20.2	16.0	28.6	15.6	23.0	30.6	26.2	18.0	27.0	23.6	40.6
Valine	107.8	118.0	98.2	122.2	107.8	85.2	146.2	124.6	143.0	142.8	147.8	84.4	137.2
Tyrosine	19.0	29.2	41.2	28.0	28.4	25.0	30.6	26.4	29.6	28.6	30.8	17.0	15.6
Trimethylamine N-oxide	3.6	9.6	4.8	2.2	2.6	2.0	1.8	3.2	3.4	3.6	3.6	3.2	8.6
Threonine	89.6	96.8	91.4	111.0	121.0	91.8	129.6	85.8	114.4	90.2	122.0	53.6	46.0
Taurine	80.4	34.6	45.2	16.0	11.2	31.8	45.2	64.6	37.6	71.0	17.8	32.4	37.6
Succinate	3.8	2.8	5.0	4.6	2.2	3.4	4.6	4.2	2.6	3.4	2.8	3.4	1.2
Serine	67.6	68.4	39.6	73.2	73.4	58.0	98.6	77.0	68.8	59.8	47.4	62.8	46.2
Pyruvate	8.8	11.4	4.4	11.8	8.8	24.8	8.4	16.6	9.6	6.2	17.2	10.8	6.0
Proline	118.8	126.4	94.0	114.8	145.0	71.6	97.8	88.6	117.0	107.8	90.2	35.8	91.0
Phenylalanine	26.2	31.4	27.4	22.2	26.8	26.6	30.8	34.0	20.6	35.4	20.6	41.6	34.4
Ornithine	23.0	29.8	27.8	29.0	39.8	20.8	32.8	26.6	26.8	28.6	33.6	21.0	19.6
O-Acetylcarnitine	5.6	6.2	4.6	2.6	5.1	6.2	6.6	7.2	7.2	5.0	6.4	6.2	6.4
N,N-Dimethylglycine	3.2	2.4	2.6	2.4	4.6	2.4	2.0	2.4	2.6	2.2	4.4	2.4	3.0
Methionine	13.6	11.8	10.4	17.2	19.6	13.4	19.4	11.8	16.2	16.2	11.0	10.6	6.8
Methanol	122.4	127.6	72.6	148.6	128.0	111.6	116.8	156.0	96.6	152.6	153.2	117.0	69.4
Mannose	31.6	49.6	55.4	60.8	84.0	44.2	61.6	44.6	60.8	31.2	52.2	50.8	42.8
Lysine	57.0	69.4	65.0	61.2	75.6	36.8	66.8	49.0	78.0	75.8	52.8	37.0	34.4
Leucine	61.0	68.6	49.0	63.2	65.0	45.6	76.2	74.2	76.8	74.8	78.0	48.8	81.8
Lactate	1434.0	1072.4	2911.4	1105.6	839.6	695.8	1261.6	2047.0	703.2	1651.4	884.8	1408.8	946.6
Isoleucine	31.2	42.2	30.8	31.2	33.0	40.6	52.6	42.0	36.4	37.8	57.2	17.2	46.6
Isobutyrate	2.8	3.4	2.8	0.0	3.4	1.8	2.8	4.0	2.6	2.0	4.6	2.6	2.0
Inosine	19.6	16.2	0.0	0.0	0.0	0.0	16.4	14.8	0.0	8.4	0.0	9.0	16.6
Hypoxanthine	16.2	14.8	0.0	0.0	0.0	0.0	14.2	14.6	0.0	13.8	0.0	31.6	0.0
Histidine	40.2	37.8	53.6	38.4	39.4	27.8	53.6	34.6	57.4	43.0	40.0	23.8	24.4
Glycine	253.6	158.4	102.2	128.0	315.0	126.6	141.4	170.4	149.2	152.8	97.0	98.6	76.8
Glycerol	70.2	88.0	67.8	81.6	119.2	96.4	81.8	100.2	68.0	54.2	71.6	63.2	68.2
Glutamine	214.6	238.0	305.8	189.6	334.6	183.8	285.6	200.4	280.0	355.4	209.8	165.8	215.4
Glutamate	50.0	13.8	32.4	54.4	50.8	27.4	57.4	68.6	36.8	64.4	50.2	58.8	70.0
Glucose	1625.6	2429.8	1992.8	2996.6	2438.8	2609.0	2758.0	1949.8	2198.8	1950.4	3807.0	1014.8	2258.6
Dimethylamine	3.6	5.8	4.4	6.6	13.8	3.6	3.6	4.0	0.0	2.4	8.2	3.8	4.4
Dimethyl sulfone	9.2	9.0	8.8	8.6	8.4	8.4	8.2	8.0	7.6	7.4	7.2	7.0	6.8
Creatinine	33.2	41.6	27.2	24.4	43.6	23.2	24.4	30.4	41.8	28.6	47.8	46.8	50.8
Creatine	15.6	44.2	10.8	21.6	16.2	7.6	23.8	44.0	15.0	12.8	18.0	12.4	18.4
Citrate	50.0	65.0	55.2	34.4	63.2	49.6	43.0	96.4	51.0	47.6	64.2	26.4	19.8
Choline	14.6	11.7	11.8	12.6	27.6	9.0	14.4	14.0	17.8	15.2	13.2	11.6	15.8
Carnitine	25.4	40.4	36.2	24.4	45.0	15.6	34.6	22.8	29.2	26.6	43.2	21.4	19.8
Betaine	17.4	5.4	21.2	18.4	27.4	12.6	20.4	10.6	17.4	20.6	26.6	18.6	15.2
Aspartate	35.6	59.2	34.2	42.0	44.4	49.2	69.8	45.0	44.4	25.8	61.8	39.0	25.2
Asparagine	32.4	32.8	22.8	29.0	41.4	34.6	32.0	25.4	21.4	40.8	24.2	24.8	15.8
Alanine	171.4	168.6	149.0	158.6	211.2	128.4	227.0	200.8	243.6	250.4	150.0	109.2	163.0
Acetone	51.8	39.2	25.0	16.2	50.6	35.0	31.4	68.4	23.2	36.6	35.4	78.2	26.6
Acetoacetate	23.0	51.4	17.8	17.8	12.6	114.6	34.4	51.6	12.8	16.6	32.8	40.6	14.2
3-Hydroxybutyrate	80.0	142.0	35.8	27.2	47.6	295.6	87.0	155.4	37.0	24.6	45.8	133.6	27.6
Acetate	33.4	21.2	85.6	28.8	181.4	19.2	37.8	27.4	21.0	61.2	22.0	53.0	25.2
2-Aminobutyrate	18.2	5.2	12.8	6.8	9.2	7.8	23.6	11.0	17.0	14.6	25.0	11.6	12.4
Pt.	13	14	15	16	17	18	19	20	21	22	23	24	25

Pt.	26	27	28	29	30	31	32	33	34	35	36	37	38
2-Aminobutyrate	7.8	16.8	11.6	15.2	7.0	15.8	10.4	3.4	13.4	11.8	20.2	2.6	24.8
Acetate	41.6	27.0	51.0	24.2	42.4	38.4	53.6	25.8	48.2	30.4	273.2	33.0	38.8
3-Hydroxybutyrate	54.8	21.0	32.4	26.6	32.0	109.6	51.0	43.8	217.4	11.6	29.0	30.2	54.0
Acetoacetate	17.6	10.8	15.6	16.6	3.4	37.8	4.4	26.4	61.4	6.8	12.8	14.0	26.4
Acetone	23.2	7.2	26.0	13.0	37.6	27.6	67.0	40.8	88.2	7.4	93.6	12.4	31.4
Alanine	141.4	168.8	263.4	201.2	131.0	179.0	177.2	70.0	120.2	168.8	156.6	134.0	175.8
Asparagine	26.4	29.2	46.6	33.2	22.8	35.8	36.0	17.4	25.2	16.2	31.6	24.0	32.8
Aspartate	44.0	28.8	58.8	33.2	23.2	57.6	52.4	38.8	18.6	38.8	36.8	16.4	37.0
Betaine	24.4	34.8	13.6	23.4	13.4	14.2	15.2	8.4	13.2	8.8	19.2	17.6	23.2
Carnitine	31.6	47.4	48.2	43.6	21.6	48.4	21.6	18.8	18.8	12.6	23.2	20.6	21.0
Choline	10.4	18.0	13.4	12.6	10.0	16.4	7.8	7.0	10.0	8.0	13.0	9.4	11.7
Citrate	39.4	35.8	37.2	43.8	34.8	51.4	29.2	32.2	25.4	15.8	42.2	39.8	60.8
Creatine	12.0	23.0	20.6	17.0	12.0	38.4	35.4	10.8	9.6	26.6	16.6	8.2	24.4
Creatinine	34.0	28.2	23.8	35.2	17.0	15.8	26.0	19.8	18.6	18.4	28.4	23.2	28.2
Dimethyl sulfone	6.6	6.6	6.4	6.4	6.4	6.2	6.2	6.0	5.8	5.8	5.6	5.6	5.6
Dimethylamine	5.6	6.2	8.0	3.6	1.8	4.6	3.6	2.0	3.6	1.4	8.2	0.0	3.6
Glucose	2102.4	2297.2	1882.8	2735.2	1298.8	2424.4	1126.4	837.2	1457.0	2262.2	2067.8	1436.4	4821.2
Glutamate	21.0	36.8	45.0	63.2	53.0	45.0	90.6	33.6	55.0	32.4	37.4	48.2	67.8
Glutamine	273.6	261.8	201.8	262.2	196.8	247.2	209.6	166.8	207.6	189.2	297.0	183.6	222.2
Glycerol	56.6	48.8	66.8	59.6	46.8	108.8	76.0	39.2	58.0	28.2	54.8	46.6	40.8
Glycine	105.6	124.0	178.8	124.6	125.2	180.0	131.4	89.2	113.6	90.4	105.6	212.4	98.6
Histidine	51.0	56.4	52.6	51.0	21.6	50.4	24.8	14.0	8.0	26.0	47.2	39.4	39.2
Hypoxanthine	0.0	0.0	0.0	0.0	15.2	0.0	27.2	25.2	27.0	0.0	0.0	0.0	0.0
Inosine	0.0	0.0	0.0	0.0	11.6	0.0	0.0	29.0	8.8	0.0	0.0	0.0	0.0
Isobutyrate	2.8	2.8	2.2	2.6	1.0	0.0	1.4	1.2	1.2	1.0	2.6	0.0	3.8
Isoleucine	33.4	27.0	31.6	22.8	22.6	32.8	36.2	15.8	47.8	19.2	38.4	12.0	35.4
Lactate	519.8	641.6	2354.8	651.8	850.8	859.6	1138.4	2786.4	1885.8	549.6	733.2	3134.4	803.2
Leucine	72.8	57.0	56.8	46.4	40.4	54.0	67.6	30.8	55.4	26.8	69.4	31.6	78.8
Lysine	61.6	59.4	59.2	65.0	28.0	50.6	69.2	40.2	43.8	35.8	73.2	52.0	62.4
Mannose	36.8	38.0	48.0	41.6	18.6	49.0	22.2	18.0	25.4	32.2	50.6	39.0	104.2
Methanol	103.8	99.2	58.4	121.6	120.2	78.4	340.4	72.0	111.2	45.2	62.6	70.6	179.8
Methionine	13.4	11.8	15.0	10.2	7.6	14.8	17.2	8.2	13.6	10.8	12.8	9.6	11.2
N,N-Dimethylglycine	2.4	2.8	2.8	3.4	1.2	2.6	1.8	1.0	1.4	1.2	3.8	2.4	2.4
O-Acetylcarnitine	5.2	7.0	5.0	6.6	4.2	7.0	3.6	3.0	4.4	2.8	5.1	3.4	7.0
Ornithine	22.0	35.4	20.0	25.0	24.0	31.0	68.4	21.8	29.4	12.8	29.2	23.6	19.4
Phenylalanine	22.2	17.6	19.2	13.8	23.2	25.4	38.8	19.8	28.4	18.0	23.0	20.0	17.6
Proline	146.0	121.2	185.2	98.2	74.6	154.0	63.2	52.0	79.8	76.0	119.4	151.0	123.4
Pyruvate	8.2	6.4	59.4	10.2	3.8	32.8	4.0	26.2	7.0	27.0	10.8	24.8	37.8
Serine	58.2	50.8	65.4	60.8	54.8	67.0	121.2	48.8	70.6	38.4	60.0	57.2	55.4
Succinate	2.0	3.0	2.6	1.8	2.8	4.4	3.0	1.4	2.6	1.4	2.8	2.4	2.4
Taurine	28.8	47.2	31.6	42.6	60.2	27.2	123.0	28.0	50.4	32.0	41.0	14.4	55.6
Threonine	129.8	123.2	144.8	98.4	55.0	144.8	77.0	42.0	55.2	51.4	94.6	84.8	86.8
Trimethylamine N-oxide	2.2	4.0	3.8	2.4	1.6	4.4	3.2	2.8	3.4	5.6	1.0	2.0	3.2
Tyrosine	32.0	27.2	28.4	23.4	16.0	31.6	36.0	21.6	22.4	17.8	32.0	31.6	33.0
Valine	129.8	110.0	112.0	122.4	75.6	93.2	112.4	60.8	94.0	76.8	132.4	61.6	162.0
myo-Inositol	10.0	18.4	14.2	27.0	17.8	17.6	15.4	11.0	23.2	10.8	14.8	13.6	28.0

Pt.	39	40	41	42	43	44	45	46	47	48	49	50	51
myo-Inositol	18.8	18.0	27.6	28.8	15.4	18.6	18.4	14.4	15.6	19.2	16.4	13.4	36.4
Valine	70.8	103.2	120.4	147.2	113.2	118.8	118.0	129.2	93.4	111.2	92.4	125.2	93.2
Tyrosine	28.0	16.0	36.8	26.6	28.0	26.1	26.0	37.0	32.0	28.0	20.4	25.0	22.8
Trimethylamine N-oxide	5.8	1.4	4.8	3.8	1.0	3.9	4.2	3.8	1.4	3.0	3.6	1.6	2.2
Threonine	105.6	74.0	73.6	122.4	94.0	63.9	88.0	105.2	106.4	68.2	58.8	123.2	88.6
Taurine	52.4	47.8	102.4	40.2	44.6	35.4	56.4	17.4	42.0	64.0	53.6	50.6	25.8
Succinate	2.6	2.8	4.8	3.0	0.8	2.7	3.2	2.4	2.8	3.2	2.6	2.6	1.0
Serine	32.6	69.6	86.4	60.0	56.8	29.1	70.0	74.2	73.2	62.4	56.6	79.8	58.2
Pyruvate	34.8	4.8	33.6	9.0	7.8	5.1	16.4	10.8	5.0	6.0	3.0	4.4	10.6
Proline	86.6	69.8	85.2	206.6	118.8	88.8	70.2	156.2	60.6	76.0	55.6	101.0	78.4
Phenylalanine	15.8	22.4	45.6	18.2	24.6	23.7	25.4	26.2	24.0	26.6	23.2	23.0	20.6
Ornithine	27.2	25.4	56.0	26.4	20.4	26.7	37.6	27.6	23.2	33.4	18.8	33.6	35.0
O-Acetylcarnitine	5.1	6.4	4.8	5.8	6.4	4.5	6.2	3.6	4.2	5.2	5.4	6.0	4.8
N,N-Dimethylglycine	4.4	2.4	8.4	3.6	4.8	2.4	2.4	4.4	2.6	1.8	2.0	2.2	2.6
Methionine	12.4	8.4	11.6	12.0	13.0	10.5	12.2	14.8	13.6	10.4	8.6	16.2	11.2
Methanol	26.8	66.6	142.4	157.8	121.6	58.5	86.6	80.4	156.8	166.8	93.6	138.6	148.0
Mannose	33.4	15.0	0.0	48.8	47.4	31.2	16.0	55.8	39.0	13.8	24.6	32.6	30.4
Lysine	57.8	40.2	60.0	69.4	55.2	48.0	52.8	68.2	61.6	42.2	59.6	68.2	59.2
Leucine	41.4	61.4	76.8	74.0	75.8	48.3	78.6	76.2	49.8	60.4	51.2	66.8	59.6
Lactate	7274.2	1193.8	4012.0	892.6	810.4	533.4	1586.0	927.8	979.2	2322.6	731.6	796.4	1246.0
Isoleucine	25.2	31.2	35.2	44.2	37.2	25.8	33.2	28.6	22.8	34.0	26.4	35.6	25.6
Isobutyrate	2.8	1.8	3.2	4.4	3.0	1.8	2.8	2.2	2.6	2.0	2.8	1.4	2.2
Inosine	0.0	5.6	0.0	6.2	0.0	0.0	18.0	0.0	22.8	16.2	3.8	0.0	26.6
Hypoxanthine	0.0	18.8	56.8	0.0	0.0	0.0	27.2	0.0	14.0	25.4	10.8	11.4	15.2
Histidine	27.8	27.8	52.0	46.8	48.0	27.3	51.8	42.6	48.6	42.4	19.0	83.6	46.2
Glycine	91.0	167.2	196.0	162.8	122.6	101.1	127.8	266.4	151.6	115.8	95.8	183.2	138.0
Glycerol	39.0	50.2	110.8	74.8	48.0	31.8	89.4	71.6	68.8	54.4	60.6	72.4	78.0
Glutamine	120.4	232.6	146.4	253.6	272.2	190.2	189.6	295.2	232.6	199.6	213.4	350.8	212.6
Glutamate	78.8	51.2	129.2	59.2	58.8	26.4	47.8	33.2	36.4	66.0	43.2	54.4	44.2
Glucose	1456.8	833.6	1564.0	2675.2	2044.6	1879.5	1015.4	2374.8	1407.4	377.6	1223.6	1865.2	2194.2
Dimethylamine	3.6	3.6	1.6	8.6	0.0	3.6	5.6	4.6	3.4	2.4	3.6	4.8	2.6
Dimethyl sulfone	5.6	5.6	5.6	5.4	5.2	5.1	5.0	4.4	4.4	4.4	4.4	4.2	4.2
Creatinine	15.8	16.8	25.2	32.8	31.6	20.7	27.0	26.4	29.8	30.2	18.8	23.4	81.8
Creatine	7.2	24.6	9.2	22.0	18.8	12.6	23.6	15.0	23.8	16.0	12.8	12.0	8.0
Citrate	39.8	52.0	47.2	55.2	30.6	21.0	48.0	42.8	51.6	14.2	50.2	45.6	37.2
Choline	11.7	9.4	16.0	18.6	14.6	9.6	12.0	12.8	9.6	14.8	10.6	10.0	9.4
Carnitine	25.4	16.4	11.2	40.0	25.8	21.0	24.6	26.0	35.4	20.4	19.2	40.6	26.0
Betaine	19.0	8.4	14.4	18.4	20.4	10.8	8.6	13.6	15.0	27.4	9.4	19.8	6.8
Aspartate	41.0	20.4	51.2	50.4	33.6	38.8	41.2	41.8	34.2	39.2	22.4	28.6	27.4
Asparagine	22.4	26.6	49.6	44.2	25.4	25.2	31.0	28.6	42.6	22.2	20.6	32.0	24.8
Alanine	104.2	121.0	264.8	236.6	241.8	75.6	99.8	171.8	169.0	172.2	120.0	164.8	129.8
Acetone	11.2	68.2	13.6	20.2	36.0	48.6	84.6	75.2	16.0	23.2	53.8	50.2	146.0
Acetoacetate	5.0	5.2	16.6	17.0	20.6	11.4	13.2	52.2	13.0	4.8	8.4	40.8	55.0
3-Hydroxybutyrate	48.8	178.0	41.2	40.8	28.2	36.9	72.2	146.4	36.4	21.4	80.6	59.0	208.0
Acetate	57.2	48.0	77.2	30.0	33.8	108.9	145.4	62.8	38.8	19.2	66.0	62.0	264.6
2-Aminobutyrate	8.6	10.8	10.8	14.8	16.4	8.7	15.0	17.4	9.2	9.2	12.2	16.2	14.4

Metabolite	52	53	54	55	56	57	58	59	60	61	62	63	64
myo-Inositol	0.0	19.2	9.2	15.0	29.0	24.0	23.8	6.4	55.4	15.0	17.8	15.2	15.8
Valine	98.8	110.4	63.2	110.2	139.6	97.6	129.8	49.0	153.8	107.0	141.8	106.2	138.0
Tyrosine	18.2	35.8	12.2	22.2	34.6	29.8	37.8	15.0	38.0	20.8	25.0	18.6	29.4
Trimethylamine N-oxide	1.2	4.4	2.8	4.2	3.2	2.0	2.0	0.6	3.0	3.4	1.6	1.8	2.2
Threonine	110.4	95.8	45.2	79.6	90.2	68.6	98.6	21.2	116.4	46.0	100.0	133.4	71.0
Taurine	23.8	33.8	18.2	58.6	66.8	40.8	46.2	12.6	133.6	29.2	36.8	41.0	21.2
Succinate	3.4	1.6	1.4	2.4	3.4	2.0	2.4	0.4	5.0	1.6	2.4	2.6	2.6
Serine	61.0	45.8	34.8	66.4	88.6	51.0	66.8	50.0	119.4	46.8	57.4	65.6	59.8
Pyruvate	11.8	13.4	15.2	4.8	11.8	9.4	32.2	34.4	98.0	4.8	5.0	9.2	7.4
Proline	78.0	94.6	62.2	63.6	80.2	72.2	149.8	49.6	116.8	74.0	87.0	98.2	69.8
Phenylalanine	22.2	38.8	15.8	30.0	41.8	36.0	30.8	12.8	51.2	23.2	23.4	13.0	45.0
Ornithine	16.6	23.2	19.2	24.2	43.0	25.8	29.6	11.2	60.6	27.6	27.2	21.2	45.0
O-Acetylcarnitine	5.1	4.0	3.0	4.0	5.2	4.4	3.8	1.6	10.6	5.2	6.4	5.1	3.6
N,N-Dimethylglycine	2.4	1.6	1.4	1.4	1.8	1.4	3.6	2.5	2.0	2.6	3.4	2.2	2.2
Methionine	10.6	13.4	7.0	10.0	20.2	14.8	17.2	8.6	21.6	9.6	10.8	10.0	17.0
Methanol	145.0	83.8	66.2	86.0	61.8	100.2	115.8	150.2	256.6	65.0	122.4	156.2	189.4
Mannose	50.4	39.8	25.4	33.4	21.6	18.8	33.8	39.0	39.0	25.4	53.6	27.8	33.4
Lysine	40.0	71.6	32.4	52.4	86.4	43.0	65.0	20.2	95.6	41.4	59.8	45.2	77.0
Leucine	68.0	68.8	25.6	55.2	78.8	53.6	65.2	26.0	115.6	55.4	57.6	46.4	78.2
Lactate	613.0	2159.8	319.4	902.0	2303.0	1420.4	888.0	8637.6	5047.6	699.8	592.6	498.4	1672.6
Isoleucine	31.2	32.4	15.8	27.4	36.8	34.0	37.4	16.8	49.6	29.8	38.8	22.0	52.6
Isobutyrate	2.6	2.8	0.8	1.4	3.2	1.2	4.8	1.2	3.4	2.2	2.6	2.4	1.8
Inosine	0.0	16.2	0.0	6.0	8.2	13.6	0.0	21.6	0.0	16.8	0.0	0.0	24.2
Hypoxanthine	0.0	30.0	0.0	9.6	41.4	19.8	0.0	22.0	41.2	10.4	0.0	0.0	28.8
Histidine	42.8	46.6	33.8	19.8	41.6	23.2	41.6	20.4	66.4	25.2	49.2	43.2	17.4
Glycine	109.8	132.4	71.6	124.0	115.8	109.0	143.2	80.4	212.8	90.8	116.4	183.4	135.4
Glycerol	79.4	43.8	35.2	46.6	73.8	74.8	48.6	18.2	171.2	39.8	55.4	77.0	82.6
Glutamine	222.8	187.2	151.4	198.2	284.6	210.4	188.8	88.8	228.6	248.0	264.2	253.0	190.2
Glutamate	44.2	32.0	26.4	48.2	84.6	51.2	35.0	48.2	181.4	44.0	37.2	22.6	64.2
Glucose	2111.6	1311.4	2303.8	1376.4	871.8	966.4	2100.2	472.2	2055.8	1115.6	2169.6	1998.6	1419.8
Dimethylamine	6.4	3.4	1.2	2.4	2.4	3.6	3.4	1.6	2.8	1.8	3.6	3.6	3.6
Dimethyl sulfone	4.0	4.0	4.0	3.8	3.8	3.8	3.6	3.6	3.6	3.6	3.4	3.4	3.4
Creatinine	22.6	30.6	20.8	15.6	31.2	25.0	25.8	17.8	39.6	23.8	32.8	25.2	35.6
Creatine	21.6	10.2	8.8	17.0	35.2	18.6	9.6	2.2	26.4	8.0	13.4	16.2	22.8
Citrate	54.4	33.6	23.0	24.2	51.0	15.2	54.8	29.8	46.2	45.8	42.2	38.8	21.0
Choline	11.7	12.8	5.6	8.0	15.2	7.4	15.4	4.0	20.6	11.2	13.4	11.7	9.2
Carnitine	25.4	30.8	17.6	17.2	18.0	23.6	41.2	8.8	25.2	16.2	29.8	25.4	18.0
Betaine	16.0	18.8	9.4	14.8	14.0	7.4	21.0	6.4	15.2	23.8	23.0	8.6	15.2
Aspartate	38.4	38.4	38.8	22.6	25.0	21.6	33.8	38.8	52.6	13.6	38.8	25.6	40.8
Asparagine	29.0	24.4	22.4	27.0	27.0	23.2	28.8	28.8	43.2	22.4	40.4	28.4	34.0
Alanine	140.4	192.0	108.8	140.2	178.2	168.8	218.0	59.0	265.0	133.8	132.2	164.0	163.8
Acetone	73.8	16.4	29.6	47.8	71.4	13.6	16.6	19.4	61.2	50.0	82.8	11.4	31.4
Acetoacetate	81.0	5.8	4.4	6.4	5.6	16.6	16.4	18.6	16.6	9.8	39.8	12.4	5.4
3-Hydroxybutyrate	144.6	21.6	14.4	61.6	84.0	18.6	20.0	37.4	99.4	48.8	156.8	41.2	22.4
Acetate	28.8	38.8	45.4	41.6	49.8	34.2	21.0	20.8	94.4	48.8	31.8	25.4	51.2
2-Aminobutyrate	15.8	8.0	9.2	10.8	15.0	16.4	17.6	6.0	13.4	10.0	20.0	14.4	7.6
Pt.	52	53	54	55	56	57	58	59	60	61	62	63	64

myo-Inositol	14.2	19.4	11.0	23.6	14.4	14.2	17.4	17.5	20.0	6.4	16.8
Valine	109.8	104.6	125.6	169.4	112.8	77.8	78.6	92.2	83.8	121.0	78.8
Tyrosine	20.2	24.2	20.4	38.0	24.6	24.0	18.2	12.5	20.8	29.6	15.6
Trimethylamine N-oxide	3.2	4.3	1.0	3.6	2.2	2.8	1.8	2.2	1.2	2.6	1.6
Threonine	56.4	73.0	104.4	122.6	119.2	68.4	36.6	55.2	101.4	141.2	32.0
Taurine	44.2	58.8	16.6	50.2	50.0	61.6	48.6	78.2	51.4	45.8	27.6
Succinate	1.4	1.7	3.4	2.2	4.2	2.2	3.2	3.4	2.4	2.2	2.6
Serine	47.4	47.8	40.4	75.2	86.0	61.2	42.8	57.8	67.6	75.0	39.8
Pyruvate	5.0	10.1	6.2	9.4	6.0	30.2	10.8	25.0	22.8	8.2	4.4
Proline	74.6	53.8	68.0	222.2	133.0	76.0	44.8	86.6	92.2	133.4	44.8
Phenylalanine	20.8	31.9	23.8	28.6	22.0	24.4	23.8	28.1	27.0	26.2	26.6
Ornithine	22.0	28.1	29.2	37.0	26.8	26.4	14.0	15.4	24.0	34.0	19.8
O-Acetylcarnitine	5.2	3.1	4.4	4.4	5.1	3.2	5.0	6.2	2.8	6.2	3.6
N,N-Dimethylglycine	2.4	1.4	3.2	2.8	3.0	4.8	1.4	1.9	3.0	2.8	1.2
Methionine	9.2	11.3	18.0	16.8	16.6	14.8	10.8	9.4	10.0	18.2	7.4
Methanol	63.0	90.2	56.2	224.2	148.2	52.2	75.6	84.5	136.6	64.8	117.0
Mannose	24.0	37.2	35.6	31.4	38.2	43.6	25.4	42.7	68.6	77.4	25.8
Lysine	41.2	54.5	57.4	86.6	60.6	32.4	27.2	31.9	29.4	67.6	33.2
Leucine	53.6	48.5	70.8	78.8	57.8	29.2	37.6	45.8	54.4	70.8	40.4
Lactate	739.8	1075.2	489.0	1170.4	609.8	654.2	1547.6	1231.0	898.6	772.6	1672.6
Isoleucine	29.4	30.0	42.0	42.0	32.0	30.6	17.6	26.4	38.6	47.0	20.0
Isobutyrate	2.0	1.7	3.6	5.4	1.8	1.6	1.2	1.7	3.0	1.8	1.4
Inosine	16.4	6.5	0.0	15.6	0.0	0.0	29.2	0.0	0.0	0.0	6.0
Hypoxanthine	9.8	14.4	0.0	15.4	0.0	0.0	25.4	0.0	0.0	0.0	23.6
Histidine	26.0	23.5	39.2	61.0	50.6	13.6	8.6	15.4	23.2	36.4	17.4
Glycine	92.0	145.4	113.6	141.6	175.0	106.2	74.4	97.2	119.0	156.8	124.2
Glycerol	38.4	37.9	79.8	43.6	81.8	27.8	44.8	200.2	111.6	88.0	65.6
Glutamine	208.6	227.0	210.4	289.2	264.4	166.4	128.8	183.6	154.0	294.8	174.4
Glutamate	38.2	51.6	48.2	35.8	40.4	76.2	34.4	51.6	36.0	35.6	43.0
Glucose	1082.0	1847.8	2067.0	2774.6	2095.2	1699.4	1424.4	1944.7	2294.0	2098.4	1500.4
Dimethylamine	1.6	2.9	6.8	6.8	2.8	1.6	1.6	2.9	7.4	7.0	2.4
Dimethyl sulfone	3.4	3.4	3.2	3.0	2.8	2.4	2.2	2.2	2.0	1.8	1.8
Creatinine	24.2	18.2	34.4	36.8	23.0	12.0	19.8	17.0	32.4	34.4	19.0
Creatine	9.2	7.9	14.4	20.8	32.4	14.8	12.8	18.0	9.0	20.4	26.0
Citrate	48.4	70.1	30.0	46.2	49.8	13.8	22.8	34.8	30.8	55.6	58.4
Choline	11.2	9.4	11.4	10.6	8.0	5.2	10.2	11.0	5.0	11.0	9.2
Carnitine	17.0	11.8	28.8	32.8	31.2	20.2	19.4	18.5	17.8	29.0	19.6
Betaine	21.4	14.6	21.8	16.6	13.4	15.4	16.4	11.5	28.0	9.8	9.4
Aspartate	38.8	21.8	45.0	54.6	46.6	23.0	21.2	20.9	42.8	30.2	19.0
Asparagine	22.0	23.5	31.2	48.4	27.0	24.4	28.8	19.7	0.0	34.8	15.4
Alanine	125.6	179.8	105.2	162.6	193.6	183.6	105.0	202.6	91.4	148.8	102.0
Acetone	57.6	14.2	59.4	24.6	15.8	11.6	100.4	100.6	52.4	31.8	76.4
Acetoacetate	12.0	8.4	53.4	11.2	17.6	3.2	25.0	10.6	135.4	61.2	4.6
3-Hydroxybutyrate	49.2	20.9	53.2	26.4	39.2	8.0	54.6	143.0	415.8	94.0	64.4
Acetate	48.8	60.7	120.0	20.6	26.4	33.4	19.8	67.0	26.8	21.4	50.6
2-Aminobutyrate	12.0	12.0	15.4	9.0	18.0	11.8	11.0	10.1	4.2	11.4	7.0
Pt.	65	66	67	68	69	70	71	72	73	74	75
